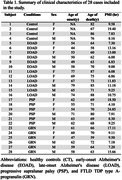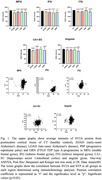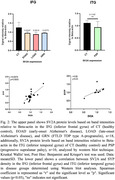# Neuropathological Mapping of Synaptic Vesicle Protein 2A in Human Brains: Ground Truth for PET Interpretation in Neurodegenerative Diseases

**DOI:** 10.1002/alz.091579

**Published:** 2025-01-03

**Authors:** Mahsa Shanaki Bavarsad, Salvatore Spina, William Seeley, Gil D. Rabinovici, Lea T. Grinberg

**Affiliations:** ^1^ Memory and Aging Center, Weill Institute for Neurosciences, University of California, San Francisco (UCSF), San Francisco, CA USA; ^2^ Memory and Aging Center, UCSF Weill Institute for Neurosciences, University of California San Francisco, San Francisco, CA USA; ^3^ Memory & Aging Center, Department of Neurology, University of California in San Francisco, San Francisco, CA USA; ^4^ Memory and Aging Center, University of California, San Francisco, San Francisco, CA USA; ^5^ Memory and Aging Center, UCSF Weill Institute for Neurosciences, University of California, San Francisco, San Francisco, CA USA

## Abstract

**Background:**

Synaptic loss, a key indicator of cognitive decline in neurodegenerative diseases, lacks a clinical biomarker, but emerging PET‐scan tracers targeting synaptic vesicle protein 2A (SV2A) show promise. The current understanding of regional changes in neurodegenerative disorders and the distribution of SV2A in the human brain is quite limited. This knowledge gap presents challenges when assessing the feasibility of using SV2A tracers in therapeutic applications. To assess the potential of SV2A tracers in diagnosis and therapy monitoring and enhance SV2A PET data interpretation, we quantified SV2A and synaptophysin (SYP) density in postmortem human brain tissue. We included subjects with different neurodegenerative conditions and healthy controls (CT) and mapped five susceptible regions.

**Method:**

Histological slides (8µm) of 28 cases (Table 1) underwent immunohistochemistry SV2A and synaptophysin. Slides were scanned using a calibrated Zeiss Axioscan. Synaptic density was quantified using the Zen software in 40 cortical ROIs (20 white & 20 gray matter) per area/case. Values were normalized by white matter intensity. Moreover, we used frozen tissue from a subset of the same cases and areas to perform Western blot (WB) using the same antibodies against SV2A and (SYP), plus Beta‐actin. Intensity values were measured by using Image J software.

**Result:**

The immunohistochemical analysis revealed decreased SV2A density across all diseases, exhibiting distinctive regional and disease‐specific patterns (Fig. 1). However, the correlation between SV2A and synaptophysin density was weak (Fig. 1). Western blot analysis similarity showed a distinctive reduction of SV2A with regional and disease‐specific patterns. The correlation with synaptophysin was moderate (Fig. 2).

**Conclusion:**

In conclusion, our findings demonstrate reduced SV2A density and protein levels in disease categories corresponding to specific regional vulnerabilities, corroborating SV2A as a reliable marker of brain tissue integrity. Nevertheless, the observed correlation between SV2A and synaptophysin changes was less robust than anticipated, prompting questioning into whether SV2A changes accurately reflect synaptophysin alterations. Additional studies comparing the utility of both markers in predicting clinical decline, metabolic shifts, and the progression of neurodegeneration are warranted.